# Clinical and anatomical characteristics of mandibular angle fractures: a 10-year retrospective monocentric cohort study

**DOI:** 10.3389/froh.2026.1910889

**Published:** 2026-07-27

**Authors:** Simon Kienberger, Stefan Krennmair, Matthias Angerer, Gerald Krennmair, Michael Malek, Lukas Postl

**Affiliations:** 1 Department of Oral and Maxillofacial Surgery, Kepler University Hospital GmbH, Johannes Kepler University Linz, Linz, Austria; 2 Medical Faculty, Johannes Kepler University Linz, Linz, Austria; 3 Department of Oral and Maxillofacial Surgery and Facial Plastic Surgery, LMU Medizin, LMU University Hospital, Ludwig-Maximilians-Universität München, Munich, Germany; 4 Head of Department of Prosthodontics, Sigmund Freud University Vienna, Vienna, Austria

**Keywords:** biomechanics, craniofacial morphology, fracture patterns, mandibular angle fracture, trauma mechanism, treatment complexity

## Abstract

**Introduction:**

Mandibular angle fractures represent a distinct subgroup of mandibular fractures with unique anatomical and biomechanical characteristics. However, factors influencing fracture patterns and treatment complexity remain incompletely understood. This study aimed to characterize the clinical, anatomical, and morphological features of mandibular angle fractures and to identify factors associated with fracture behavior.

**Methods:**

This retrospective monocentric cohort study included 115 patients treated for mandibular angle fractures at a tertiary referral center between January 2015 and December 2025. Demographic, fracture related, radiological, morphological, dental, and treatment variables were retrospectively collected and analyzed to evaluate factors associated with fracture patterns, treatment complexity, and clinical outcomes.

**Results:**

Of 115 patients, 104 (90.4%) were male and 11 (9.6%) were female. Female patients were significantly older than male patients (*p* < 0.001). Contralateral fracture patterns predominated among multiple fractures, most commonly involving the paramedian region. Pathological fractures were significantly associated with isolated fractures (*p* = 0.020) and occurred exclusively in patients aged ≥30 years (*p* < 0.001). Dentition status differed significantly between isolated and multiple fractures (*p* = 0.031). Morphological parameters, particularly anterior facial height and the posterior to anterior facial height (PFH/AFH) ratio, were significantly associated with fracture multiplicity and treatment complexity. Third molar status was significantly associated with treatment complexity (*p* = 0.010), whereas treatment modality differed significantly between age groups (*p* = 0.020). No significant association was observed between trauma mechanism and fracture pattern or treatment complexity.

**Conclusion:**

Mandibular angle fractures appear to be primarily influenced by anatomical and biomechanical characteristics rather than trauma mechanism alone. Craniofacial morphology, age, and dental status may contribute to fracture patterns and treatment complexity, supporting a more individualized assessment of mandibular angle fractures.

## Introduction

Mandibular fractures represent one of the most common injuries of the facial skeleton and account for a substantial proportion of maxillofacial trauma cases worldwide (1, 2). Within the mandible, the angle region is consistently reported as one of the most frequently affected sites, alongside the condyle and parasymphysis (3). Epidemiological studies have demonstrated that mandibular angle fractures account for approximately 20%–30% of all mandibular fractures, making them a clinically highly relevant entity (4, 5).

The increased susceptibility of the mandibular angle to fracture is attributed to a combination of anatomical and biomechanical factors, including a reduced cross-sectional bone area, the frequent presence of impacted or partially erupted third molars, and the influence of masticatory muscle forces (6, 7). Owing to these characteristics, mandibular angle fractures are associated with comparatively high complication rates and remain a challenging entity in oral and maxillofacial surgery (8). Management continues to be debated, particularly with regard to fixation strategies and the management of third molars (9–11).

Several anatomical, morphological, and clinical factors have been proposed to influence fracture behavior, including craniofacial morphology, trauma mechanism, age, dentition status, and third molar status (12). However, despite the growing body of literature on mandibular fractures, their relative contribution to fracture patterns and treatment complexity in mandibular angle fractures remains incompletely understood. In particular, the interaction between craniofacial morphology, patient characteristics, and fracture behavior has not been systematically investigated in this distinct fracture entity (13, 14).

Beyond their anatomical and biomechanical characteristics, mandibular angle fractures present important clinical challenges. They are associated with relatively high rates of postoperative complications, including infection, malocclusion, hardware failure, and delayed bone healing (15, 16). Surgical management remains demanding, with treatment decisions depending not only on fracture configuration but also on patient related characteristics, dentition status, third molar position, and anticipated fixation requirements. Identifying clinical factors associated with fracture patterns, treatment complexity, and postoperative outcomes may improve preoperative risk assessment, facilitate individualized treatment planning, and ultimately optimize patient outcomes (15–19).

Therefore, the aim of the present study was to comprehensively characterize the clinical, anatomical, and morphological features of mandibular angle fractures and to identify factors associated with fracture patterns, treatment complexity, and postoperative complications within a 10 year retrospective monocentric cohort. By integrating demographic, morphological, fracture related, treatment related, and clinical variables, this study sought to improve the understanding of mandibular angle fracture behavior and to support more individualized diagnostic assessment and treatment planning.

## Materials and methods

### Study design and setting

This retrospective monocentric cohort study was conducted at a tertiary referral center for oral and maxillofacial surgery (Kepler University Hospital, Linz, Austria). All patients treated for mandibular fractures between January 1, 2015, and December 17, 2025, were screened for eligibility. Clinical and radiological data were retrieved from electronic medical records and institutional imaging databases.

### Participants

All patients diagnosed with mandibular fractures who were treated at the Department of Oral and Maxillofacial Surgery between January 1, 2015, and December 17, 2025, were screened for eligibility. The only inclusion criteria were the presence of a mandibular angle fracture and treatment within the predefined study period. Mandibular angle fractures were initially identified by reviewing the corresponding medical records and surgical reports of all 632 patients with mandibular fractures and were subsequently confirmed by retrospective evaluation of the available preoperative computed tomography (CT) scans, where fractures were anatomically classified according to their location within the mandibular angle. Patients with mandibular fractures not involving the angle region were excluded. No patients were excluded because of incomplete clinical or radiological data, previous treatment, recurrent fractures, or any other reason. A total of 632 patients with mandibular fractures were screened, of whom 115 patients met the inclusion criteria and were included in the final analysis. The remaining 517 patients were excluded solely because they did not present with a mandibular angle fracture. Patient inclusion and exclusion are summarized in Supplementary Material 1.

### Ethical approval

This study was approved by the ethics committee of the Johannes Kepler University Linz (approval no. 1485/2025; February 4, 2026). The requirement for informed consent was waived due to the retrospective design.

### Variables

The primary objective was to identify anatomical, morphological and clinical factors associated with fracture characteristics and treatment complexity. Sex was evaluated as an exploratory descriptive variable. A comprehensive set of demographic, anatomical, radiological, and clinical variables was collected. Demographic data included sex. Anatomical variables comprised ramus height, ramus length, minimum and maximum ramus width, as well as the gonial and antegonial angles.

Fracture related variables included fracture location and classification, the presence of third molars within the fracture line, Gregory Pell classification (class and position), and the presence of pathological fractures (20). As no standardized classification of treatment complexity for mandibular angle fractures is currently available, treatment complexity was operationally defined for the purposes of the present study based on fixation requirements. Simple fixation was defined as the use of one to two plates, whereas complex fixation was defined as the use of three or more plates. This pragmatic categorization was introduced solely for exploratory analytical purposes and was intended to provide clinically interpretable treatment groups while maintaining statistically meaningful subgroup sizes. The number of fixation plates was used as a pragmatic analytic parameter for the extent of surgical stabilization required but should not be interpreted as a validated or direct measure of intraoperative treatment complexity or surgical severity. This operational definition was established exclusively for the present study and should not be interpreted as a validated classification system. Radiological variables included anterior facial height (AFH), posterior facial height (PFH), and the PFH to AFH ratio.

Clinical variables included mechanism of injury, occurrence of complications, and treatment modality. Dental status, tooth extractions, and the presence of teeth within the fracture line were additionally recorded.

**Figure 1:**
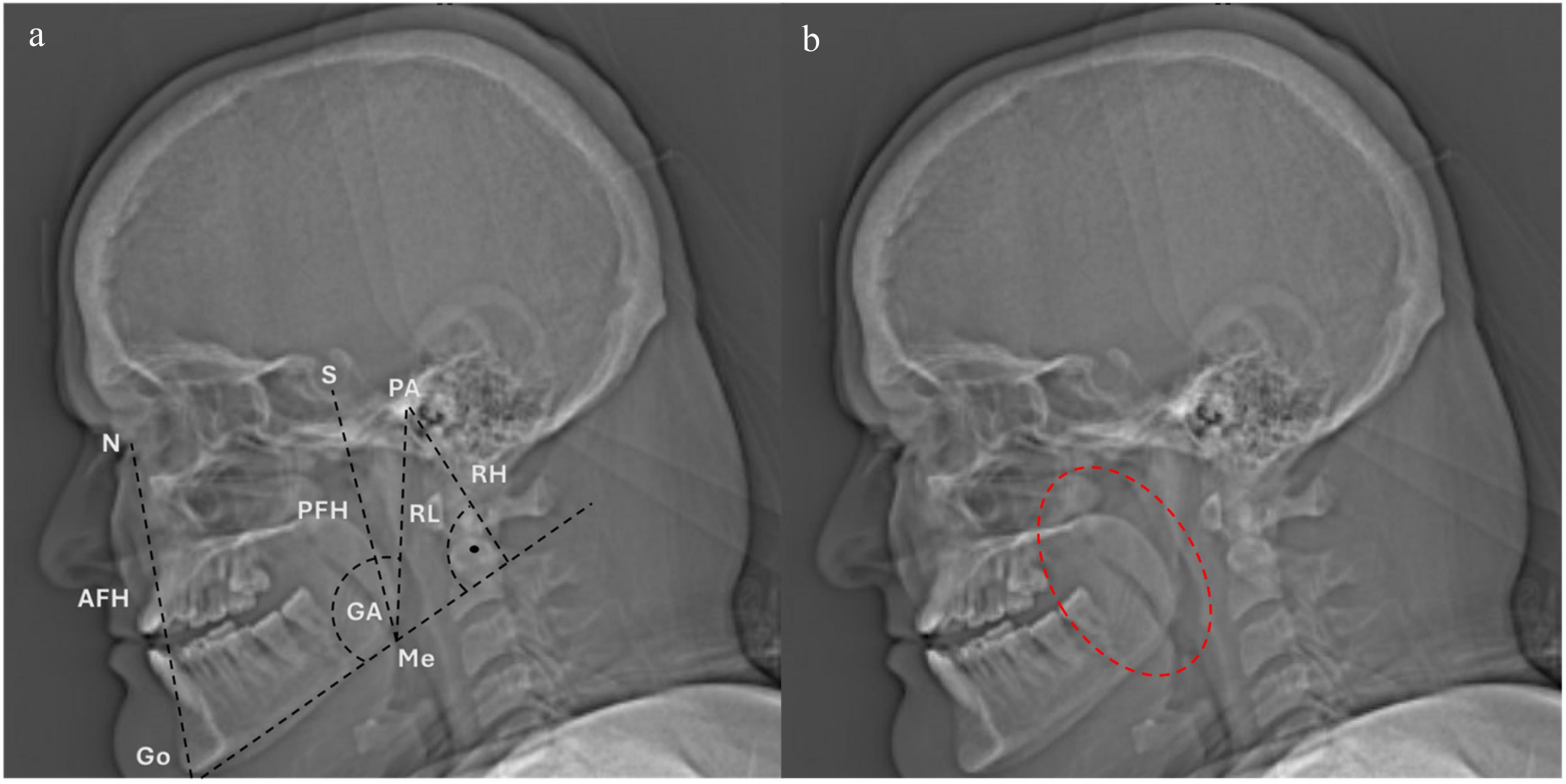
Radiological assessment and morphometric measurements of mandibular angle fractures. (**a**) Representative lateral cephalometric scout radiograph illustrating the anatomical landmarks and morphometric measurements used for craniofacial assessment. Anterior facial height (AFH) was measured as the linear distance between Nasion (N) and Menton (Me), whereas posterior facial height (PFH) was measured between Sella (S) and Gonion (Go). Ramus height (RH) was defined as the distance from the extended inferior mandibular border to the tip of the processus articularis (PA), and ramus length (RL) as the distance between the tip of the processus articularis and Gonion (Go). The gonial angle (GA) was measured between tangents to the posterior border of the ramus and the inferior border of the mandibular body. The PFH/AFH ratio was subsequently calculated from the measured facial height values. For clarity and to avoid visual overcrowding, the antegonial angle is not displayed. (**b**) Representative illustration highlighting the mandibular angle region (red dashed circle), including an example of a representative mandibular angle fracture location. The highlighted region served as the anatomical reference for fracture.

Treatment related variables comprised the surgical approach including the use of transbuccal techniques, the number of fractures, and the presence of ipsilateral or contralateral combination fractures (Figure 1).

### Data sources and measurement

Data were retrospectively extracted from electronic medical records and radiographic imaging. Fracture classification and anatomical measurements were based on preoperative imaging including CT scans. CT imaging was performed using standardized acquisition parameters (100 kV, 413 mAs).

Clinical variables including complications, treatment strategies, and mechanisms of injury were obtained from surgical reports and clinical documentation. When multiple imaging modalities were available, CT imaging was preferentially used for morphometric assessment.

### Radiologic measurement protocol

All imaging based variables, including fracture classification, fracture pattern, Gregory Pell classification, pathological fracture assessment, third molar status, and morphometric measurements, were retrospectively evaluated on multiplanar preoperative CT reconstructions using the institutional imaging software CITRIX with digital linear and angular measurement tools. Anatomical landmarks and predefined radiological criteria, as described in the Variables section, were established *a priori* and applied consistently throughout the analysis. All assessments were performed independently by a single board-certified oral and maxillofacial surgeon. To ensure measurement consistency, a subset of cases was reassessed, confirming intraobserver reliability with no relevant measurement deviation observed. As all imaging assessments were performed by a single examiner, interobserver variability could not be assessed.

Ramus height was defined as the linear distance from the extended inferior mandibular border to the tip of the processus articularis. Ramus length was measured as the distance between the tip of the processus articularis and the gonion. Maximum and minimum ramus width were defined as the greatest and smallest transverse dimensions of the mandibular ramus.

The gonial angle was defined as the angle between a tangent to the posterior border of the ramus and a tangent to the inferior border of the mandibular body. The antegonial angle was measured at the antegonial notch using tangential lines along the inferior mandibular border. Both measurements were based on previously described cephalometric definitions (21).

Anterior facial height was measured on frontal CT reconstructions as the distance between Nasion and Menton, whereas posterior facial height was assessed on sagittal reconstructions as the distance between Sella turcica and Gonion according to Gandhi et al. (22). For illustrative purposes, the anatomical landmarks and morphometric measurements are presented on a representative lateral cephalometric radiograph (Figure 1) as the anatomical landmarks can be visualized more clearly than on CT reconstructions. All measurements were performed on preoperative CT reconstructions.

### Data analysis

The study size was determined by the number of eligible patients within the study period, and no *a priori* sample size calculation was performed due to the retrospective design.

Given the exploratory nature of this study, analyses were primarily descriptive and hypothesis-generating rather than confirmatory.

Continuous variables were reported as mean values or, in the case of non-normally distributed data, as median with interquartile range, while categorical variables were expressed as absolute frequencies and percentages.

Data distribution was assessed using the Shapiro–Wilk test. Group comparisons were performed using the independent samples t-test for normally distributed variables and the Mann–Whitney U test for non normally distributed variables. Categorical variables were analyzed using the chi-square test or Fisher's exact test as appropriate.

For exploratory subgroup analyses, patients were stratified into two age groups (<30 years and ≥30 years). As no validated age threshold exists for mandibular angle fractures, the selected cutoff was chosen as a pragmatic methodological approach. The categorization was intended to facilitate clinically interpretable comparisons by reducing age-related heterogeneity within the exploratory subgroups and avoiding the combination of younger adults with substantially older patients into the same analysis group. This dichotomization was performed exclusively for exploratory subgroup analyses and should not be interpreted as representing a biologically validated threshold.

Subgroup analyses were conducted to evaluate associations between fracture patterns, treatment complexity, age groups, and patient-related characteristics, including craniofacial morphology, trauma mechanism, dentition status, third molar status, and sex. Owing to the exploratory nature of the study and the limited sample size, particularly the low number of female patients, no confirmatory multivariable analyses were performed. Therefore, sex was considered an exploratory variable and interpreted descriptively throughout the analyses. A two sided *p* value of less than 0.05 was considered statistically significant.

This study was conducted in accordance with the Strengthening the reporting of observational studies in epidemiology (STROBE) guidelines (23).

## Results

### Study population

A total of 115 patients with mandibular angle fractures were included, comprising 104 (90.4%) male and 11 (9.6%) female patients. The mean age of the study population was 39.4 ± 19.0 years. Female patients were significantly older than male patients (62.1 ± 25.0 vs. 36.8 ± 16.0 years, *p* < 0.001).

Isolated mandibular angle fractures predominated, whereas multiple fractures occurred less frequently. Among patients with multiple fractures, contralateral fracture patterns predominated (90.7%). No significant sex related differences were observed regarding fracture characteristics (Table 1).

**Table 1:** Demographic, clinical, morphological, and dental characteristics of the study population.

Variable	Total (*n* = 115)	Male (*n* = 104)	Female (*n* = 11)	*p*-value
Demographics				
Age (years, mean ± SD)	39.4 ± 19.0	36.8 ± 16.0	62.1 ± 25.0	<0.001
Fracture Characteristics				
Fracture Number, *n* (%)				0.478
Single Fracture	68 (59.1)	61 (58.7)	7 (63.6)	
Double Fracture	43 (37.4)	40 (38.5)	3 (27.3)	
Triple Fracture	4 (3.5)	3 (2.9)	1 (9.1)	
Multiple fracture distribution), *n* (%)				0.565
Contralateral fractures	39 (90.7)	36 (90.0)	3 (100)	
Ipsilateral fractures	4 (9.3)	4 (10)	0 (0.0)	
Pathologic fractures, *n* (%)	8 (7.0)	8 (7.7)	0 (0.0)	0.6*
Morphological Characteristics				
Ramus Height, mm	51.6 ± 8.1	52.4 ± 7.9	44.8 ± 6.3	0.001
Ramus Length, mm	64.3 ± 6.9	65.1 ± 6.3	55.8 ± 6.7	<0.001
Min. Ramus width, mm	27.5 ± 5.4	27.5 ± 5.4	27.9 ± 5.4	0.927
Max. Ramus width, mm	37.1 ± 4.8	37.2 ± 4.4	36.9 ± 8.1	0.221
Gonial Angle,°	125.4 ± 8.3	125.3 ± 8.4	126.2 ± 8.2	0.379
Antegonial Angle,°	161.3 ± 7.6	161.3 ± 7.8	161.8 ± 6.0	0.972
AFH, mm	119.2 ± 9.9	120.0 ± 9.9	111.4 ± 5.9	0.002
PFH, mm	78.3 ± 8.7	79.4 ± 8.1	67.4 ± 7.6	<0.001
PFH/AFH Ratio	0.66	0.66	0.61	0.010
Dental Status				
G.P. Classification Class, *n* (%)				0.239
Class 1	27 (32.5)	26 (33.8)	1 (16.7)	
Class 2	29 (34.9)	25 (32.5)	4 (66.7)	
Class 3	27 (32.5)	26 (33.8)	1 (16.7)	
G.P. Classification Position, *n* (%)				0.851
Position A	37 (44.0)	35 (44.9)	2 (33.3)	
Position B	25 (29.8)	23 (29.5)	2 (33.3)	
Position C	22 (26.2)	20 (25.6)	2 (33.3)	
Dentition Status, *n* (%)				0.240
Fully dentate	82 (71.3)	76 (73.1)	6 (54.5)	
Partially dentate	25 (21.7)	22 (21.1)	3 (27.2)	
edentulous	8 (7.0)	6 (5.8)	2 (18.2)	
Third Molar in Fracture Line, *n* (%)	66 (57.4)	61 (58.7)	5 (45.5)	0.532

Data are presented as mean ± standard deviation (SD) for continuous variables and as frequency (n) and percentage (%) for categorical variables.

Min, minimal; Max, maximal; PFH, posterior face height; AFH, anterior face height; G.P., Gregory Pell Classification.

Male patients exhibited significantly greater ramus height, ramus length, anterior facial height, posterior facial height, and PFH/AFH ratio than female patients (Table 1). No significant differences were observed for ramus width, gonial angle, antegonial angle, dentition status, Gregory Pell classification, or the presence of a third molar in the fracture line (Table 1). A participant flowchart illustrating patient selection is provided in Supplementary Material 1.

### Fracture patterns and trauma mechanism

Among the 43 patients with multiple mandibular fractures, the paramedian region was the most frequent accompanying fracture site (51.1%). Associated fractures occurred predominantly contralateral to the mandibular angle fracture (90.7%), whereas ipsilateral fracture combinations were uncommon (Tables 2, 3). Falls (32.2%) and interpersonal violence (28.7%) were the predominant trauma mechanisms, followed by other causes (27.0%), sports related injuries (7.0%), and road traffic accidents (5.2%). Fracture multiplicity varied descriptively across trauma mechanisms; however, no statistically significant association was observed (Table 4).

**Table 2:** Fracture patterns in double fractures.

Variable	Body	paramedian	Condyles	Angle	Ramus	Total
Total Double Fractures, *n* (%)	8 (18.6)	22 (51.1)	7 (16.2)	5 (11.6)	1 (2.3)	43
Male Double Fracture Combination, *n* (%)	8 (20.0)	20 (50.0)	7 (17.5)	4 (10.0)	1 (2.5)	40
Female Double Fracture Combination, *n* (%)	0	2 (66.7)	0	1 (33.3)	0	3

Anatomical distribution of associated fracture locations in patients with double mandibular fractures.

**Table 3:** Fracture distribution relative to the angle fracture.

Localisation of Second Fracture, *n* (%)	Contralateral (*n* = 39, 90.7%)	Ipsilateral (*n* = 4, 9.3%)
Paramedian	20 (51.2)	2 (50.0)
Body	6 (15.3)	2 (50.0)
Condyles	7 (17.9)	0 (0.0)
Angle	5 (12.8)	0 (0.0)
Ramus	1 (2.5)	0 (0.0)

Distribution of the second fracture site in relation to angle fractures. Percentages are calculated within the contralateral (*n* = 39) and ipsilateral (*n* = 4) groups.

**Table 4:** Distribution of fracture causes according to sex and fracture type.

Sex	Cause Category	Fracture Type			Total	*p*-value
		Single	Double	Triple		
Male, *n* (%)						0.130
	Falls	16 (53.3)	13 (43.3)	1 (3.3)	30	
	Interpersonal Violence	16 (51.6)	14 (45.2)	1 (3.2)	31	
	Road traffic accidents	1 (20)	3 (60)	1 (20)	5	
	Sport injuries	5 (62.5)	3 (37.5)	0 (0.0)	8	
	Other causes	23 (76.7)	7 (23.3)	0 (0.0)	30	
	Total	61	40	3	104	
Female, *n* (%)						n.a.
	Falls	4 (57.1)	2 (28.8)	1 (14.3)	7	
	Interpersonal Violence	1 (50.0)	1 (50.0)	0	2	
	Road traffic accidents	1 (100)	0 (0.0)	0 (0.0)	1	
	Sport injuries	0 (0.0)	0 (0.0)	0 (0.0)	0	
	Other causes	1 (100.0)	0 (0.0)	0 (0.0)	1	
	Total	7	3	1	11	
Total, *n* (%)						0.235
	Falls	20 (54.1)	15 (40.5)	2 (5.4)	37	
	Interpersonal Violence	17 (51.5)	15 (45.5)	1 (3.0)	33	
	Road traffic accidents	2 (33.3)	3 (50)	1 (16.7)	6	
	Sport injuries	5 (62.5)	3 (37.5)	0 (0.0)	8	
	Other causes	24 (77.4)	7 (22.6)	0 (0.0)	31	

Data are presented as frequency (n) and percentage (%). Percentages are calculated within each trauma mechanism category. Statistical analysis was not performed for female patients because of the small sample size.

n.a., not applicable.

### Treatment characteristics and postoperative complications

Most patients underwent ORIF combined with intermaxillary fixation and were treated with simple fixation (1 to 2 plates). No significant sex-related differences were observed regarding treatment complexity, treatment modality, or fixation plate type (Table 5). Overall, 13 postoperative complications were recorded during follow-up, most commonly infection and hardware-related complications.

**Table 5:** Treatment characteristics of the study population.

Variable	Total (*n* = 115)	Male (*n* = 104)	Female (*n* = 11)	*p*-value
Treatment Complexity, *n* (%)				0.487
Simple	99 (86.1)	89 (85.6)	10 (90.0)	
Complex	10 (8.7)	10 (9.6)	0 (0.0)	
Type of Therapy, *n* (%)				0.465
ORIF	19 (16.5)	16 (15.4)	3 (27.3)	
ORIF + IMF	90 (78.3)	83 (79.8)	7 (63.6)	
Plate Type used in Surgery, *n* (%)				0.240
≤ 4-hole plate	91 (79.1)	82 (78.9)	9 (81.8)	
min. 1 plate with 4 or more holes	18 (15.7)	17 (16.4)	1 (9.1)	
Conservative Treatment	6 (5.2)	5 (9.1)	1 (4.8)	

Data are presented as counts and percentages.

ORIF, open reduction and internal fixation; IMF, intermaxillary fixation; min, minimal use of.

### Factors associated with fracture pattern, treatment complexity and age

Comparison of isolated and multiple mandibular angle fractures demonstrated that pathological fractures occurred exclusively in patients with isolated fractures (*p* = 0.020). In addition, dentition status differed significantly between isolated and multiple fracture patterns (*p* = 0.031), whereas no further significant associations were observed (Table 6).

**Table 6:** Comparison of demographic, clinical, and treatment characteristics of patients with single and multiple mandibular angle fractures.

Variable	Single Fracture	Multiple Fracture	*p*-value	Effect Size
Pathological, *n* (%)	8 (100)	0 (0)	0.020	0.227
Dentation Status, *n* (%)			0.031	0.301
Fully dentate	43 (53.8)	37 (46.3)		
Partially dentate	19 (82.6)	4 (17.4)		
edentulous	6 (75)	2 (25)		
Cause of Fracture, *n* (%)			0.239	0.250
Falls	20 (57.1)	15 (42.9)		
Interpersonal Violence	17 (53.1)	15 (46.9)		
RTA	2 (40.0)	3 (60.0)		
Sport Injuries	5 (62.5)	3 (37.5)		
Other causes	24 (77.4)	7 (22.6)		
Treatment Complexity, *n* (%)			0.476	0.116
Simple	57 (59.4)	39 (40.6)		
Complex	6 (66.7)	3 (33.3)		
Conservative Treatment	5 (83.3)	1 (16.7)		
Treatment, *n* (%)			0.090	0.224
ORIF	15 (79.0)	4 (21.0)		
ORIF + IMF	48 (55.8)	38 (44.2)		
Conservative Treatment	5 (83.3)	1 (16.7)		
Third Molar Status, *n* (%)			0.396	0.073
Present	37 (57.8)	27 (42.2)		
Absent	29 (65.9)	15 (34.1)		

Comparison of demographic, dental, and treatment characteristics between patients with single and multiple mandibular fractures. Categorical variables are presented as counts and percentages.

ORIF, open reduction and internal fixation; IMF, intermaxillary fixation; RTA, road traffic accidents.

The exploratory treatment categorization used in the present study was significantly associated with pathological fractures (*p* < 0.001), third molar status (*p* = 0.010), and treatment modality (*p* < 0.001). Pathological fractures were more frequently assigned to the complex fixation category or managed conservatively, whereas the presence of a third molar was more frequently associated with the simple fixation category (Table 7).

**Table 7:** Comparison of clinical characteristics according to treatment modality and treatment complexity.

Variable	Treatment Complexity			*p*-value	Effect Size
	Simple	Complex	Conservative		
Pathological, *n* (%)	3 (37.5)	3 (37.5)	2 (25.0)	< 0.001	0.385
Cause of Fracture, *n* (%)				0.774	0.145
Falls	34 (91.9)	2 (5.4)	1 (2.7)		
Interpersonal Violence	26 (78.8)	4 (12.1)	3 (9.1)		
RTA	5 (83.3)	1 (16.7)	0 (0.0)		
Sport Injuries	8 (100)	0 (0.0)	0 (0.0)		
Other causes	26 (83.9)	3 (9.7)	2 (6.5)		
Treatment, *n* (%)				< 0.001	0.711
ORIF	16 (84.2)	3 (15.8)	0 (0.0)		
ORIF + IMF	83 (92.2)	7 (7.8)	0 (0.0)		
Conservative Treatment	0 (0.0)	0 (0.0)	6 (100)		
Third Molar Status, *n* (%)				0.010	0.207
Present	62 (93.9)	2 (3.0)	2 (2.0)		
Absent	34 (73.9)	8 (17.4)	4 (8.7)		

Comparison of clinical characteristics according to treatment approach. Categorical variables are presented as counts and percentages.

ORIF, open reduction and internal fixation; IMF, intermaxillary fixation; RTA, road traffic accidents.

Exploratory age subgroup analyses demonstrated that all pathological fractures occurred in patients aged ≥30 years (*p* < 0.001). Treatment modality also differed significantly between the predefined age groups, with patients aged ≥30 years more frequently undergoing ORIF alone, whereas ORIF combined with intermaxillary fixation was more common in younger patients (Table 8).

**Table 8:** Comparison of clinical characteristics according to age group (<30 years vs. ≥ 30 years).

Variable	Age group		*p*-value	Effect Size
	< 30 years	≥ 30 years		
Pathological, *n* (%)	0 (0.0)	8 (100)	< 0.001	0.312
Fracture pattern, *n* (%)			0.090	0.158
Isolated Fracture	34 (50.0)	34 (50.0)		
Multiple Fracture	31 (66.0)	16 (34.0)		
Cause of Fracture, *n* (%)			0.200	0.228
Falls	19 (51.4)	18 (48.7)		
Interpersonal Violence	23 (69.7)	10 (30.3)		
RTA	2 (33.3)	4 (66.7)		
Sport Injuries	6 (75.0)	2 (25.0)		
Other causes	15 (48.4)	16 (51.6)		
Treatment Complexity, *n* (%)			0.246	0.156
Simple	59 (59.6)	40 (40.4)		
Complex	4 (40.0)	6 (60.0)		
Conservative Treatment	2 (33.3)	4 (66.7)		
Treatment, *n* (%)			0.020	0.261
ORIF	6 (31.6)	13 (68.4)		
ORIF + IMF	57 (63.3)	33 (36.7)		

Comparison of clinical characteristics according to age group (<30 years vs. ≥ 30 years). Categorical variables are presented as counts and percentages.

ORIF, open reduction and internal fixation; IMF, intermaxillary fixation; RTA, road traffic accidents.

## Discussion

The present study provides a comprehensive characterization of mandibular angle fractures by integrating demographic, anatomical, morphological, and treatment-related characteristics. The findings suggest that fracture behavior is primarily influenced by intrinsic anatomical and biomechanical factors, whereas demographic characteristics alone appear to play a more limited role. These findings support a multifactorial concept of mandibular angle fracture development (24, 25).

One of the principal findings was the predominance of contralateral fracture patterns in patients with multiple mandibular angle fractures, with the paramedian region representing the most frequent accompanying fracture site. These findings suggest that fracture propagation follows characteristic biomechanical pathways. The mandibular angle represents a structurally vulnerable transition zone between the mandibular body and ramus, where externally applied forces are concentrated and subsequently redistributed across the mandibular arch. Consequently, stress transmission may predispose anatomically weaker regions, particularly the contralateral paramedian region, to secondary fracture formation (26, 27).

Interestingly, trauma mechanism was not significantly associated with fracture multiplicity, suggesting that fracture configuration depends not only on the external mechanism of injury but also on intrinsic anatomical and biomechanical characteristics, including mandibular geometry and stress distribution. From a clinical perspective, the predominance of contralateral fracture patterns highlights the importance of systematically evaluating the entire mandible, even when a mandibular angle fracture appears to be the primary injury (28).

The relevance of intrinsic anatomical characteristics is further underscored by the observed associations between craniofacial morphology and fracture characteristics. In the present cohort, AFH and the PFH/AFH ratio were significantly associated with fracture patterns and treatment complexity, indicating that individual craniofacial proportions may influence both fracture propagation and surgical management. Previous biomechanical and anatomical studies have demonstrated that variations in mandibular geometry affect the distribution of tensile and compressive stresses during traumatic loading, thereby influencing fracture susceptibility and propagation pathways. Although causality cannot be inferred, craniofacial morphology should be considered during diagnostic assessment and treatment planning (29, 30).

The association between fracture characteristics and the exploratory treatment categorization used in the present study further emphasizes the potential clinical relevance of these findings. Pathological fractures were more frequently assigned to the complex fixation category, whereas the presence of a third molar was more frequently associated with the simple fixation category. These observations may reflect differences in fracture configuration and bone quality rather than treatment preference alone; however, this interpretation should be considered exploratory given the operational nature of the treatment categorization. While the exploratory treatment categorization used in the present study is inevitably influenced by surgical decision making, the present findings suggest that specific anatomical and clinical characteristics may warrant consideration during preoperative planning to facilitate individualized treatment strategies (31).

Exploratory age subgroup analyses highlighted potential differences in patient characteristics among individuals with mandibular angle fractures. In the present cohort, all pathological fractures occurred in patients aged ≥30 years, and treatment modality differed significantly between the predefined age groups. As the selected age cutoff was introduced solely for exploratory subgroup analyses and does not represent a biologically validated threshold, these findings should be interpreted with caution. Nevertheless, they are in agreement with the broader literature indicating that increasing age may influence skeletal biology, fracture healing, and clinical management. In contrast, fracture pattern, trauma mechanism, and treatment complexity were not significantly associated with the predefined age groups, suggesting that these characteristics may be more strongly influenced by intrinsic anatomical and biomechanical factors than by age alone. The observation that all pathological fractures occurred in the older subgroup warrants further investigation but should be interpreted cautiously in light of the limited number of pathological fractures. Future prospective studies using larger cohorts and analyses treating age as a continuous variable are warranted to further clarify the relationship between age and mandibular angle fracture characteristics (32–34).

Although several morphological differences between male and female patients were observed, these findings should be interpreted with caution. The marked imbalance between male and female patients, together with the significant age difference between the groups, limits the ability to distinguish the independent effects of sex from those of age and other patient-related characteristics. Consequently, sex should be regarded as an exploratory descriptive variable rather than the primary determinant of fracture behavior. Instead, mandibular angle fractures appear to be influenced by a complex interaction of anatomical, morphological, and clinical factors. Future studies including larger and more balanced cohorts are required to further clarify the independent contribution of sex to mandibular angle fracture characteristics.

From a clinical perspective, the present findings emphasize the importance of an individualized approach to the assessment and management of mandibular angle fractures. The high prevalence of contralateral fracture patterns highlights the need for comprehensive radiological evaluation of the entire mandible, even when a single fracture site is initially identified. Furthermore, patient-specific anatomical characteristics, including craniofacial morphology and third molar status, may provide valuable information for preoperative planning and treatment selection. Integrating these factors into clinical decision making may contribute to more tailored treatment strategies and improved patient outcomes (35).

### Limitations

This study has several limitations that should be considered when interpreting the findings. First, its retrospective single-center design may have introduced selection bias and unmeasured confounding, thereby limiting the generalizability of the results and precluding conclusions regarding causality. Furthermore, the absence of multivariable analyses limits the identification of independent predictors and the ability to account for potential confounding factors.

Another important limitation is the relatively small sample size of specific subgroups, particularly female patients and patients with pathological fractures. Consequently, subgroup analyses involving sex should be interpreted as exploratory, and the significant age difference between male and female patients may have influenced some of the observed associations.

Finally, bone quality was not assessed directly, and all imaging-based assessments and morphometric measurements were performed by a single examiner. Although standardized assessment protocols were applied consistently throughout the study and intraobserver reliability was confirmed by reassessment of a subset of cases, interobserver variability could not be assessed. Therefore, the present findings should be regarded as hypothesis generating and require confirmation in larger prospective multicenter studies incorporating balanced cohorts, direct assessment of bone quality, and multivariable analyses.

## Conclusion

Mandibular angle fractures represent a distinct clinical entity characterized by complex anatomical, morphological, and clinical features. In the present cohort, craniofacial morphology, fracture characteristics, age, and patient-specific anatomical features were associated with fracture patterns and treatment complexity, highlighting the multifactorial nature of these injuries. These findings support an individualized approach to the assessment and management of mandibular angle fractures by integrating anatomical, morphological, and clinical characteristics into diagnostic evaluation and treatment planning. However, given the retrospective exploratory design of the present study, these associations should not be interpreted as evidence of causal relationships. Further prospective multicenter studies incorporating balanced cohorts and multivariable analyses are required to validate these findings and to clarify the independent contribution of individual patient-related factors.

## Data Availability

The datasets presented in this article are not readily available because the data supporting the findings of this study are not publicly available due to institutional data protection regulations and the sensitive nature of patient-related clinical data. Access to the data may be considered upon reasonable request and with appropriate ethical and institutional approvals. Requests to access the datasets should be directed to kienberger.simon38@gmail.com.
