# Supplementary material for: Clinical and anatomical characteristics of mandibular angle fractures: a 10-year retrospective monocentric cohort study
Source: Front Oral Health. 2026 Jul 27;7:1910889. doi: 10.3389/froh.2026.1910889 (PMC13454043; doi:10.3389/froh.2026.1910889)
Supplement: Supplementary file 1 [file Table1.docx]

Patients with mandibular angle fractures meeting the study eligibility criteria **(n = 115)**

Analyzed **(n = 115)**

Excluded **(n = 517)**

Mandibular fractures without angle involvement

No patients were excluded because of:

- Incomplete data
- Previous treatment/fractures
- Other reasons

Patients with mandibular fractures treated between
January 1, 2015, and December 17, 2025

Assessed for eligibility **(n = 632)**

Screened for eligibility by review of
medical records and surgical reports

Diagnosis confirmed by retrospective
evaluation of preoperative CT scans

**Figure 1:** Flow diagram of patient selection
